# Mapping Lifecourse Reproductive Trajectories in a Nationally Representative Cohort of U.S. Women

**DOI:** 10.1093/geroni/igaf122.4161

**Published:** 2025-12-31

**Authors:** Brianah McCoy, Lauren Gaydosh

**Affiliations:** University of North Carolina at Chapel Hill, Chapel Hill, North Carolina, United States; University of North Carolina at Chapel Hill, Chapel Hill, North Carolina, United States

## Abstract

Reproductive timing and experiences vary substantially among U.S. women, yet few studies have systematically classified reproductive life course trajectories in nationally representative, longitudinal cohorts. Using data from Waves I–V of the National Longitudinal Study of Adolescent to Adult Health (Add Health), we constructed reproductive trajectories based on age at menarche, timing of first birth, total parity, reproductive lifespan, and pregnancy-related complications. Variables were harmonized and temporally aligned to accommodate changes in survey structure across waves, and latent class analysis identified distinct reproductive patterns. These trajectories capture meaningful variation in reproductive timing and intensity that is hypothesized to be biologically relevant for chronic disease risk in midlife. This work is novel in its application to a large, nationally representative sample with rich longitudinal reproductive histories. The resulting classifications provide a foundation for ongoing research linking reproductive life course patterns to molecular indicators of aging and long-term health outcomes in women.

